# Characterization and Hepatoprotections of *Ganoderma lucidum* Polysaccharides against Multiple Organ Dysfunction Syndrome in Mice

**DOI:** 10.1155/2021/9703682

**Published:** 2021-02-03

**Authors:** Yiwen Zhang, Yanbo Feng, Wenshuai Wang, Le Jia, Jianjun Zhang

**Affiliations:** College of Life Science, Shandong Agricultural University, Taian 271018, China

## Abstract

**Background:**

The liver is one of the most commonly affected organs in multiple organ dysfunction syndrome (MODS). In recent years, there have been many studies on *Ganoderma lucidum* polysaccharides (GLP), but the role of GLP in MODS is still unclear. The purpose of this work was to explore the antioxidant, anti-inflammatory, and protective effects of GLP on the liver in MODS model mice.

**Methods:**

The characteristic properties of GLP were processed by physicochemical analysis. The MODS models were successfully established with intraperitoneal injection of zymosan in Kunming strain mice. The antioxidant, anti-inflammatory, and hepatoprotective effects of GLP were processed both *in vitro* and *in vivo* by evaluating the oxidative parameters, inflammatory factors, and liver pathological observations.

**Results:**

The characterization analysis revealed that GLP was a pyranose mainly composed of glucose with the molecular weights (Mw) of 8309 Da. The experimental results proved that GLP had potential hepatoprotection possibly by improving the antioxidant status (scavenging excessive oxygen radicals, increasing the antioxidant enzyme activities, and reducing the lipid peroxide), alleviating the inflammatory response (reducing the inflammatory factor levels), and guaranteeing the liver functions.

**Conclusions:**

This research suggested that GLP had the potential to be developed as a natural medicine for the treatment of multiple organ failure.

## 1. Introduction

Multiple organ dysfunction syndrome (MODS) is a pathophysiological status, in which the functions of some organs in patients being with acute diseases cannot effectively maintain the stability of the internal environment [1]. The MODS was firstly identified in shocking humans in the 1960s, showing the properties of successfully resuscitated patients tending to die from a complex disease failure process, which was characterized by gradual and often irreversible failure of some organs [2, 3]. Essentially, it has been reported that the MODS is a physiological process of uncontrolled inflammation throughout the body [4, 5]. A large number of inflammatory factors, such as IL-6, IL-8, TNF-*α*, and COX-2, can be released after activating the non-specific immune system by shock, burn, sepsis, and other factors, eventually develop into MODS [6]. It has been reported that intraperitoneal injection of yeast polysaccharide (zymosan) could lead to peritonitis in mice, which in turn caused systemic inflammatory response, leading to lung, kidney, and liver damage, and eventually MODS [7, 8]. Therefore, we chose the method of intraperitoneal injection of yeast polysaccharide to construct the MODS model mice for further effective prevention and treatment.

*Ganoderma lucidum* is a medicinal and edible fungus with a long history more than 2,000 years in China, which has good effects used in the prevention and clinical treatments against many diseases [9, 10]. A large number of animal experiments and studies on the composition and structures have proved that *G. lucidum* has great potential in the treatment of modern diseases such as tumors, hypertension, and hyperlipidemia [11–13]. With the development of separation and purification technology, accumulated literatures have demonstrated that the *G. lucidum* polysaccharides (GLP), among the bioactive ingredients of polysaccharides, nucleosides, triterpenes, trace elements, alkaloids, and amino acids, are the most abundant substances and play essential roles in maintaining the bioactivities [14–17]. Parallelly, Wang et al. have demonstrated that *Ganoderma lucidum* polysaccharides have immune regulation and anti-tumor activity [18], and Xu et al. have studied that *Ganoderma lucidum* polysaccharides can regulate the expression of inflammatory cytokines by increasing insulin sensitivity in mice models, thereby reducing the inflammatory response [19]. Additionally, studies have shown that the activities of polysaccharides are closely related to their chemical compositions, chain conformations, and physical properties. Therefore, the structure analysis should be determined and analyzed to understand the mechanism in structure-activity relationship.

Hence, this work was designed to investigate the anti-inflammatory, antioxidant effects, and liver protections of GLP on MODS mice models, aiming to provide references on understanding the protection mechanisms and applying GLP against multiorgan failure clinically in the pharmaceutical industry. Furthermore, the physicochemical properties were also processed aiming to get the possible structure-activities relationship.

## 2. Materials and Methods

### 2.1. Materials and Chemicals

The *G. lucidum* fruiting body was provided by the Taian Academy of Agricultural Sciences (Taian, China). Kunming strain mice were purchased from Taibang Biological Products Limited Company (Taian, China). The kits used in the experiments were purchased from Jiangsu Meibiao Biological Technology Company Limited (Jiangsu, China) and Nanjing Jiancheng Bioengineering Institute (Nanjing, China).

### 2.2. The Preparation of GLP

After the fruiting body was dried naturally, it was crushed into powder by a pulverizer (Shanghai, China). The GLP was prepared by referring to the method of Huang et al. [20] with slight modification. Polysaccharide from *G. lucidum* was extracted with distilled water (90°C), centrifuged (3000 rpm, 10 min), and the precipitation was discarded. Three times volume of ethanol (95%) was added into the supernate solution, and the precipitation was left overnight at 4°C. After centrifugation at 8000 rpm for 10 min, crude polysaccharides were collected. Sevag method was used to remove the protein repeatedly until it was completely removed, and distilled water was used for dialysis for five days. GLP was obtained after vacuum freeze-drying.

### 2.3. Structural Characterization of GLP

The monosaccharide composition of GLP was determined by high-performance liquid chromatography (HPLC) reported by Zhang et al. with slight modifications [21]. The sample hydrolysate (250 *μ*L) was accurately added to the NaOH (250 *μ*L, 0.6 mol/L) solution and mixed uniformly and reacted at 70°C for 1 h. Subsequently, the mixed solution was cooled and added with 500 *μ*L of 0.3 mol/L hydrochloric acid to neutralize; then, 1 mL of chloroform was added to the vortex and centrifuged at 3000 rpm for 10 min, and the supernatant was taken. Monosaccharide composition was determined by comparison with mannose (Man), ribose (Rib), rhamnose (Rha), glucose (Glc), glucuronic acid (GlcA), galactose (Gal), galacturonic acid (GalA), xylose (Xyl), arabinose (Arab), glucosamine (GlcN), galactosamine (GalN), and fucose (Fuc).

The GLP sample (1 mg) was mixed with KBr powder and compressed into tablets, and the data was recorded by an infrared spectrometer (Nicolet IS 10) with a scanning range of 4000-400 cm^−1^.

The bond types were processed by Fourier transform infrared spectroscopy (FT-IR) analysis. The sample was dissolved in deuterated water (D_2_O), and the ^1^H and ^13^C nuclear magnetic resonance (NMR) spectra were recorded with a Bruker AVANCE III 600 spectrometer at 25°C.

The molecular weight of polysaccharide was determined by gel permeation chromatography (GLPC). Japan Shimadzu (Shimadzu) company RID-20a refractive index detector was the detector used in the experiment, and the gel column used was a TSK gel GMPWXL aqueous gel column. In the experiment, NaNO_3_ (0.1 N) was mixed with NaN_3_ (0.06%) as the mobile phase, and the narrow curve polyethylene glycol (PEG) was used as the standard curve in the experiment.

### 2.4. Animal Experiments

In this work, male Kunming mice (8 weeks old, 20 ± 2 g) were selected. All mice were subjected to standard environmental conditions with a temperature of 22 ± 1°C, a humidity of 55 ± 5%, and a 12 h dark cycle of 12 h of light. During the experiment, free access to food and water was allowed. After seven days of acclimatization, the mice were randomly divided into five groups (10 mice per group), including one normal control (NC) group, one model control (MC) group, and three dose groups treated with GLP (GLP was dissolved in distilled water). During the gavage procedure, the mice in the three dose groups were received GLP in doses of 600, 400, and 200 mg/kg, while the mice in MC and mice in NC group were given equal amount of normal saline. The gavage was administered with a syringe once daily and continued for twenty-five successive days. Ten hours after the last administration, all the mice except that in the NC groups were intraperitoneally injected with 500 mg/kg zymosan to induce MODS [22], using normal saline as control in NC groups. After 10 days of zymosan injection, the mice were sacrificed by anesthesia according to the ethical approval by Shandong Agricultural University Committee on an empty stomach and overnight without water.

Blood sample from each mouse was obtained from the retrobulbar vein and centrifuged at 14000 rpm (4°C, 10 min) to obtain the required serum. The activities of alkaline phosphatase (ALP), alanine aminotransferase (ALT), and aspartate aminotransferase (AST) and levels of triglyceride (TG), low-density lipoprotein cholesterol (LDL-C), and high-density lipoprotein cholesterol (HDL-C) were determined using an automatic biochemical analyzer (ACE, USA).

The livers were rapidly excised, weighed, and homogenized (1 : 9, *w*/*v*) in phosphate-buffered solutions (0.2 mol/L, pH 7.4). After centrifugation at 3000 rpm for 20 min, the supernatants were stored at 4°C for further biochemical analysis. The superoxide dismutase (SOD) activities, catalase (CAT) activities, and malondialdehyde (MDA) contents were determined strictly according to the method of the kits. The ELISA kit double antibody sandwich method was used to detect the levels of tumor necrosis factor-*α* (TNF-*α*), interleukin-6 (IL-6), interleukin-1*β* (IL-1*β*), phenyl glycidyl ether 2 (PGE2), and cyclooxygenase-2 (COX-2) according to kits instructions.

The fresh livers were soaked in 10% formalin buffer and sliced in paraffin. The histopathological changes were observed under the microscope after staining with hematoxylin-eosin (HE).

### 2.5. *In Vitro* Antioxidant Capacities Analysis

The GLP was diluted with gradient deionized water, adjusting to different concentrations of 0 to 1000 *μ*g/mL. The method for the determination of scavenging hydroxyl radical ability (-OH) was slightly modified with reference to Smironf [23]. Ferrous sulfate (1 mL, 9 mmol/L), salicylic acid (1 mL, 9 mmol/L), polysaccharide solutions (1 mL), and hydrogen peroxide (1 mL, 0.03%) were sequentially added to the test tube and mixed evenly. After 30 min of water bath at 37°C, the absorbance at 510 nm was measured, and the scavenging abilities was calculated by formula (1).

(1) $$\begin{array}{c} \text{Scavenging abilities }\left(\%\right)=\left(A_{0}-A_{1}\right)/A_{0}\times 100, \end{array}$$

where *A*_0_ was the absorbance of the blank, and *A*_1_ was the absorbance of the polysaccharide samples or BHT.

The experiments referred to the method of Li et al. to determine the reducing power with slight modifications [24]. A sample solution (1 mL), 2.5 mL of phosphate buffer (pH 6.6, 0.2 mol/L), and potassium ferricyanide solution (1 ml, 1%, *w*/*v*) were sequentially added to the test tube and mixed. The test tube was placed in a 50°C water bath and reacted for 20 min. Trichloroacetic acid (2.5 mL, 10%) and ferric chloride solution (0.1%, *w*/*v*) were added to the test tube and reacted at room temperature for 10 min, and the absorbance at 700 nm was measured. Distilled water was used instead of polysaccharide solution in a blank tube; other conditions were the same.

The methods reported in the literature were slightly modified to determine the scavenging DPPH activity of GLP [25]. Polysaccharide sample solution (2 mL) of different concentrations was mixed evenly with DPPH (2 mL, 0.2 mmol/L) ethanol solution (2 mL), and ethanol was used in blank control to replace DPPH ethanol solution. The absorbance value was measured at 517 nm after the reaction of avoiding light for 30 min, and the scavenging abilities was calculated by formula (2).

(2) $$\begin{array}{c} \text{Scavenging abilities }\left(\%\right)=\left(A_{0}-A_{1}\right)/A_{0}\times 100, \end{array}$$

where *A*_0_ was the absorbance of the blank, and *A*_1_ was the absorbance of the polysaccharide samples or BHT.

### 2.6. Acute and Subchronic Toxicity Analysis

The acute and subchronic toxicity experiments were conducted with reference to Zhang et al. [26]. Seventy Kunming mice (8 weeks old, 20 ± 2 g) were randomly selected for the acute toxicity study. Thirty mice were randomly divided into a NC group and two dose groups (10 mice in each group). The mice in the dose groups were received GLP at the doses of 1000 and 1500 mg/kg, while the mice in NC groups were received isometric saline solutions. The gavage was continued for 10 days, being allowed free access to water and food. During this period, the health and death of the mice were regularly observed, and the initial and final body weights of the mice were recorded. Another forty mice were divided into four groups, including a NC group and three dose groups with 10 mice in each group. The mice in the dose groups were received GLP at doses of 900, 1200, and 1500 mg/kg, while the mice in NC groups were received isometric saline solutions. The gavage was continued for 20 days, being allowed free access to water and food. During this period, the health and death of the mice were regularly observed, and the initial and final body weights of the mice were recorded.

### 2.7. Statistical Analysis

All the measurement data were recorded as means ± standard deviation (S.D.). Significant differences between the experimental groups were determined by the one-way ANOVA followed by the Tukey Analysis (SPSS 25.0 software package, USA). *P* < 0.05 was considered to be statistically significant.

## 3. Results and Analysis

### 3.1. Structural Features Analysis

**Figure 1:**
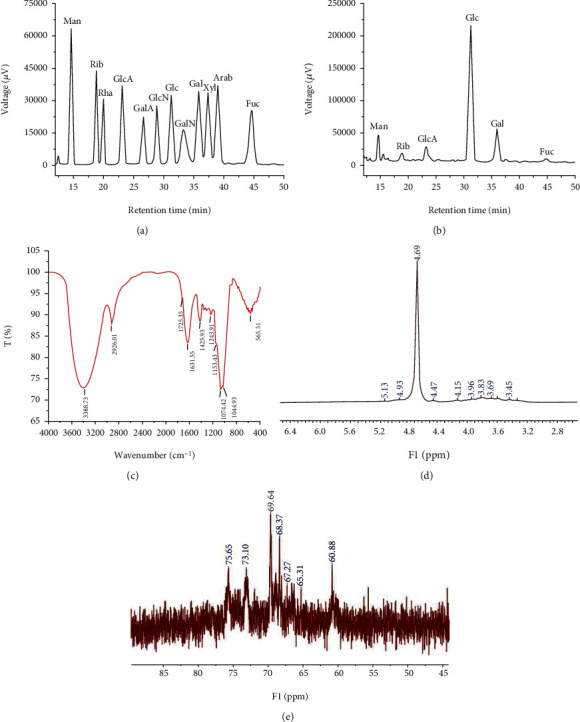
Structural characterizations of GLP. (a) High-performance liquid chromatography of 12 standard monosaccharides. (b) High-performance liquid chromatography of GLP. (c) FT-IR spectra over the range of 400-4000 cm^−1^. (d) NMR analysis of ^1^H spectra. (e) NMR analysis of ^13^C spectra. Arab: arabinose; GLP: *G. lucidum* polysaccharides; FT-IR: Fourier transform infrared spectroscopy; Fuc: fucose; Gal: galactose; GalA: galacturonic acid; GalN: galactosamine; Glc: glucose; GlcA: glucuronic acid; GlcN: glucosamine; Man: mannose; NMR: nuclear magnetic resonance; Rib: ribose; Rha: rhamnose; Xyl: xylose.

By HPLC, the monosaccharides types and contents can be determined according to the retention time of monosaccharides in standard samples. As shown in Figure 1(b), GLP contained Man, Rib, GlcA, Glc, Gal, and Fuc with the molar ratio of 7.6 : 6.0 : 9.2 : 76.5 : 18.0 : 2.5, indicating the major monosaccharide in GLP was glucose.

As display in Figure 1(c), GLP had obvious absorption peaks at 3388.73 cm^−1^ and 2926.01 cm^−1^, which were the stretching vibration absorption peaks of C-H and -OH in the molecular or intramolecular polysaccharide [27]. At 1725.35 cm^−1^ and 1631.55 cm^−1^, the telescopic vibration absorption peaks of amide-carbonyl group were observed [28]. The absorption peak at 1425.93 cm^−1^ was caused by C-H variable angle vibration, and the absorption peak at 1153.43 cm^−1^, 1074.42 cm^−1^, and 1044.93 cm^−1^ was the telescopic vibration peak of ether bond (C-O-C), which was the characteristic peak of pyranose [29, 30].

NMR plays an important role in solving the monosaccharide type and glycoside bond configuration of carbohydrate compounds. According to Figures 1(d) and 1(e), the hetero-headed proton and hetero-headed signal of GLP ^1^H NMR spectrum and ^13^C NMR spectrum were concentrated at *δ* 3.45-4.69 ppm and *δ* 60.88-75.57 ppm [31, 32]. Moreover, there was no peak at *δ* 5.4 ppm in the ^1^H spectrum and no signal at *δ* 82-88 ppm (characteristic signal of furanose) in the ^13^C spectrum, indicating that GLP was pyranose, which was consistent with the results obtained by the FT-IR analysis [32, 33]. In the ^13^C NMR spectrum, the carbon signal appears in the region of 67-73 ppm, indicating that the 6-carbon substitution of the glycosyl has occurred [34].

Gel permeation chromatography was used to determine the molecular weight of GLP. The molecular weight (Mw) of GLP was 8309 Da, and the numeral average molecular weight (Mn) was 4211 Da (Table S1).

### 3.2. Effect of GLP on Antioxidant Index *In Vivo*

**Figure 2:**
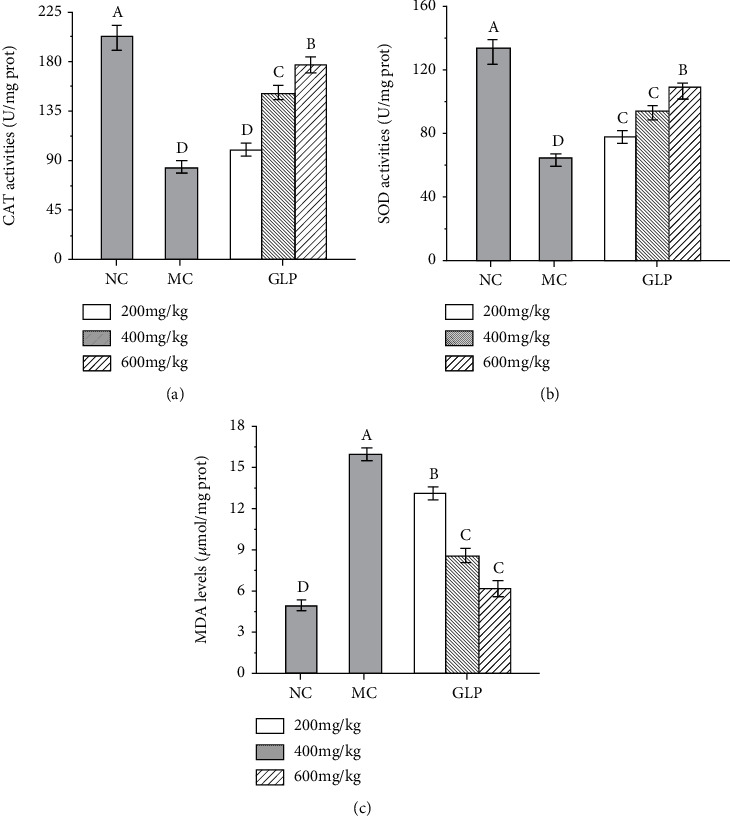
Effects of GLP on the CAT activities (a), SOD activities (b), and MDA (c) levels in the liver. The values were reported as the mean ± S.D. (*n* = 10 for each group). Significant differences between the experimental groups were determined by the one-way ANOVA followed by the Tukey Analysis. The bars with different letters are significantly different. CAT: catalase; GLP: *G. lucidum* polysaccharides; MC: Model control; MDA: malondialdehyde; NC: Normal control; SOD: superoxide dismutase.

CAT is an important enzyme that breaks down hydrogen peroxide to protect the body from oxidative stress [35]. As can be seen from Figure 2(a), the CAT activities in MC groups were significantly lower than that of NC groups (*P* < 0.05), indicating that serious oxidative stress had occurred in the liver. However, the CAT activities were significantly increased after GLP interventions, and the GLP at 600 mg/kg showed superior effects (176.40 ± 7.35 U/mg prot). SOD is an important antioxidant enzyme in the body, and the SOD activities in mice showed positive relationships with antioxidant capacity in the body [36]. As can be seen in Figure 2(b), the hepatic SOD activities in MC groups were significantly lower than that in NC groups (*P* < 0.05) indicating that certain oxidative damage had occurred in mice in the MC groups. After the intervention with GLP at 600 mg/kg, the SOD activities were increased to 107.80 ± 4.83 U/mg prot, 70.3% higher than that in MC groups. The hepatic MDA contents in MODS models were shown in Figure 2(c). When compared with that in the NC groups, the MDA contents of mice in MC groups were obviously higher (*P* < 0.05), showing that the liver of MODS mice had suffered serious oxidative damage. Interestingly, the MDA contents reached 6.10 ± 0.36 *μ*mol/mg prot treated with GLP at the dose of 600 mg/kg, suggesting that GLP can effectively reduce the production of lipid peroxide and reduce liver damage.

### 3.3. Effect of GLP on Inflammatory Factors

**Figure 3:**
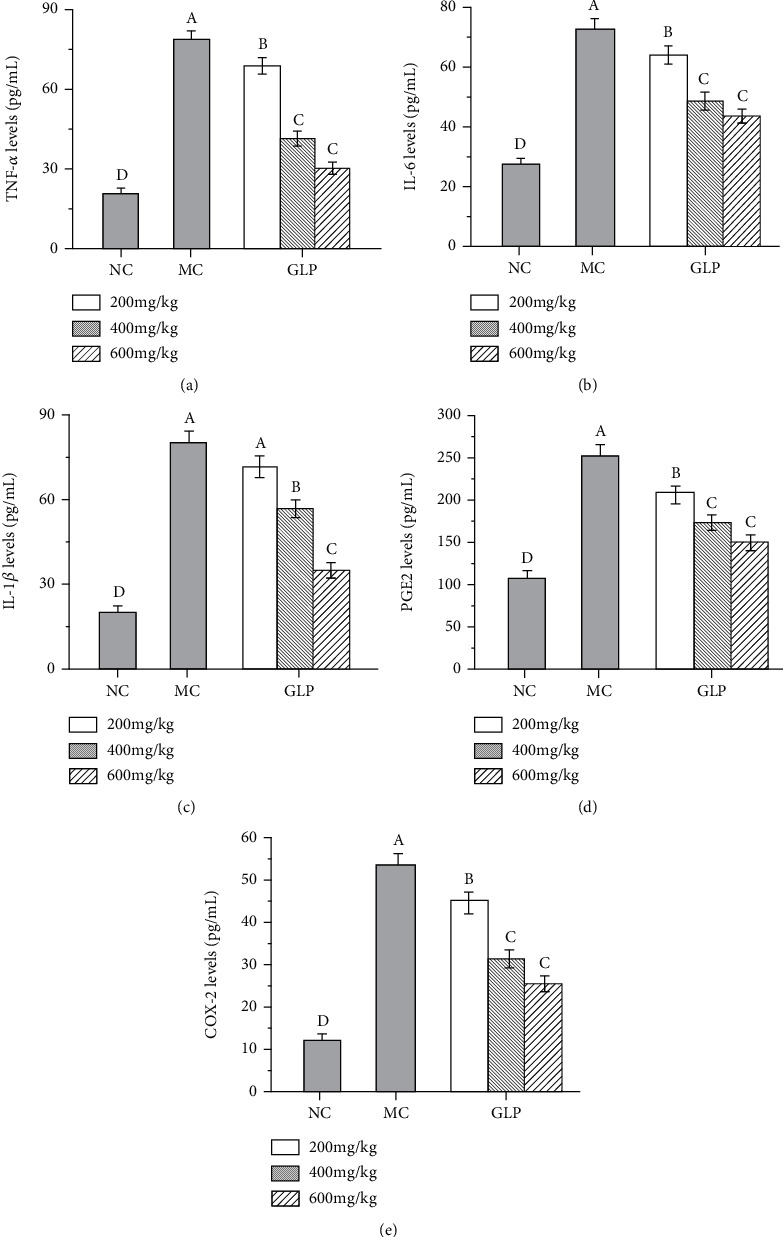
Effects of GLP on the levels of TNF-*α* (a), IL-6 (b), IL-1*β* (c), PGE2 (d), and COX-2 (e). The values were reported as the mean ± S.D. (*n* = 10 for each group). Significant differences between the experimental groups were determined by the one-way ANOVA followed by the Tukey Analysis. The bars with different letters are significantly different. COX-2: cyclooxygenase-2; GLP: *G. lucidum* polysaccharides; IL-1*β*: interleukin-1*β*; IL-6: interleukin-6; MC: model control; NC: normal control; PGE2: phenyl glycidyl ether 2; TNF-*α*: tumor necrosis factor-*α*.

To evaluate the effects of GLP on the inflammatory response in MODS mice, the levels of inflammatory factors (TNF-*α*, IL-6, IL-1*β*, PGE2, and COX-2) in the liver homogenate of MODS mice were determined by ELISA. As shown in Figure 3, the levels of TNF-*α*, IL-6, IL-1*β*, PGE2, and COX-2 of mice in the MC groups were significantly higher than those in the NC groups (*P* < 0.05), indicating that the inflammation was severe and the model was successfully constructed. Interestingly, the hepatic inflammatory cytokines contents were significantly reduced by GLP treatment in dose-dependent manners (*P* < 0.05), indicating that GLP can effectively regulate the inflammations by reducing the inflammatory cytokine levels.

### 3.4. Biochemical Assays

**Figure 4:**
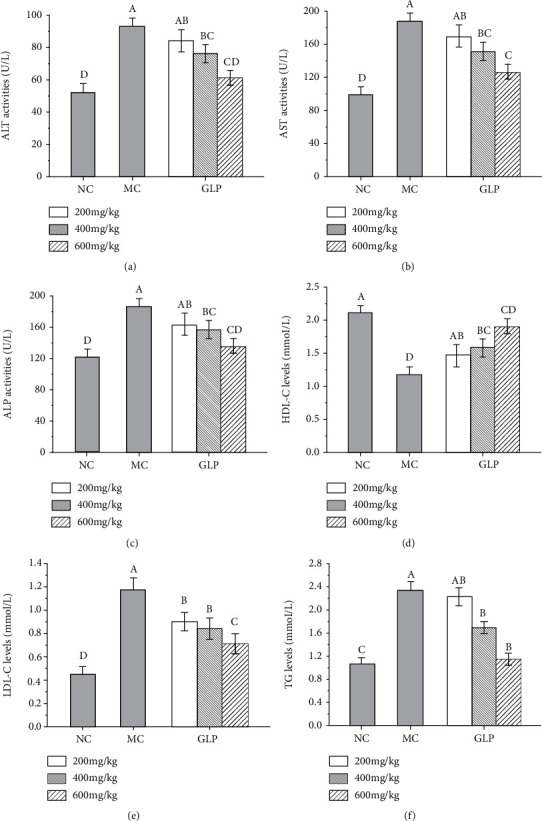
Effects of GLP on ALT activities (a), AST activities (b), ALP activities (c), HDL-C levels (d), LDL-C levels (e), and TG levels (f) in serum. The values were reported as the mean ± S.D. (*n* = 10 for each group). Significant differences between the experimental groups were determined by the one-way ANOVA followed by the Tukey Analysis. The bars with different letters are significantly different. ALT: alanine aminotransferase; AST: aspartate aminotransferase; ALP: alkaline phosphatase; GLP: *G. lucidum* polysaccharides; HDL-C: high-density lipoprotein cholesterol; LDL-C: low-density lipoprotein cholesterol; MC: model control; NC: normal control; TG: triglyceride.

The ALT, ALP, and AST activities had been recommended by the World Health Organization as the most sensitive indicators of liver injury [37]. Briefly, the ALP has been clinically proven to be too high and prone to hepatobiliary disease [38]. As shown in Figure 4, when compared with that in the NC groups, the hepatic ALT (*P* < 0.05), AST (*P* < 0.05), and ALP (*P* < 0.05) levels of mice in the MC groups were significantly increased, showing that severe liver damage had occurred in the mice. It was interesting that the activities of ALT, AST, and ALP were decreased after GLP intervention in dose-dependent manners. Especially in the high-dose groups (600 mg/kg), the ALT, AST, and ALP activities were decreased to 61.15 ± 4.61, 126.1 ± 8.55, and 135.20 ± 9.01 U/L (Figures 4(a)–4(c), *P* < 0.05). At the same time, the experimental results showed that the HDL-C and LDL-C contents of mice in the MC groups were disordered, and liver lipids were abnormal (Figures 4(d) and 4(e)). The TG contents of mice in the MC groups were increased to 2.33 ± 0.15 mmol/L (Figure 4(f), *P* < 0.05), reflecting that triglycerides were accumulated in the liver, inducing the liver damages. After GLP intervention, the pathological situations were well improved and alleviated, indicating that GLP can reduce the MODS induced liver injuries.

### 3.5. Histopathological Observation

**Figure 5:**
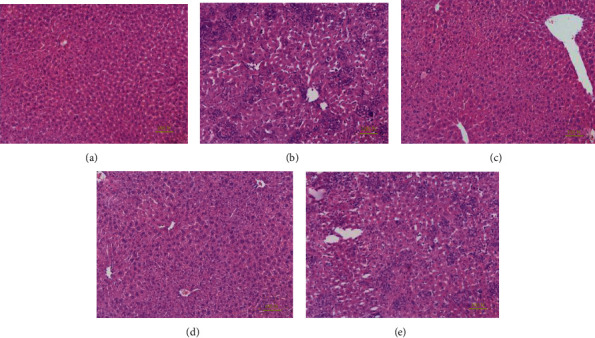
Optical micrographs of mouse liver sections by HE staining (200x magnification). (a) NC group, (b) MC group, (c) GLP at 600 mg/kg, (d) 400 mg/kg, and (e) 200 mg/kg. GLP: *G. lucidum* polysaccharides; MC: model control; NC: normal control.

In present study, the histopathological observations of the liver were performed to corroborate the evidence from biochemical analyses. The liver histopathological examination was shown in Figure 5. Observably, normal hepatic cell morphology with abundant cytoplasm, distinct nuclei, well-defined cell borders, and visible central veins can be seen in the hepatocyte from the mice in NC groups (Figure 5(a)). In contrast, hepatic sections of mice in the MC groups showed severe hepatocyte apoptosis, hepatocellular swelling, fatty accumulation, a loss of cellular boundaries, and other inflammatory changes (Figure 5(b)), indicating that the damaged liver models had been successfully established. The liver sections of the mice treated with GLP (200, 400, and 600 mg/kg) showed more or less normal cellular architectures with a loss of cellular boundaries and other inflammatory changes (Figures 5(c)–5(e)). The group treated with GLP at high levels (600 mg/kg) showed the best normal cellular architecture almost comparable to the normal control (Figure 5(c)).

### 3.6. Determination of Antioxidant Capacity of GLP *In Vitro*

**Figure 6:**
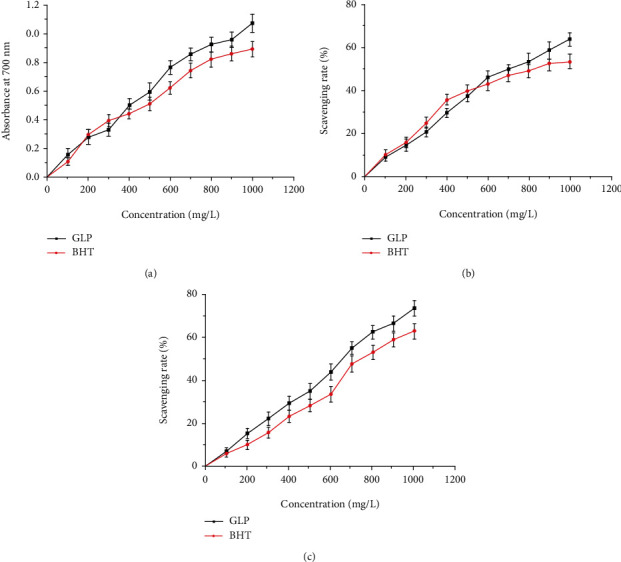
*In vitro* antioxidant activities of GLP. (a) Reducing power. (b) Scavenging abilities on hydroxyl radical. (c) Scavenging abilities on DPPH. BHT: butylated hydroxytoluene; GLP: *G. lucidum* polysaccharides.

The reducing power was one of the important indicators directly reflecting the antioxidant capacity [39]. The absorption value of the reaction system at 700 nm represented the reduction power of the antioxidant. As illustrated in Figure 6(a), the reduction power of GLP was increased with the increase of polysaccharide concentrations. When the concentration was 1000 mg/L, the maximum absorption values of GLP and BHT reached 1.07 ± 0.06% and 0.89 ± 0.05%, respectively, indicating that GLP had an obvious advantage of reduction capacity. Removal of hydroxyl radicals was essential to protect the body from damage caused by free radicals. As display in Figure 6(b), the hydroxyl radical-scavenging rate of GRP on hydroxyl radical was dose-dependent within the range of 0-1000 mg/L concentrations. At 1000 mg/L, the clearance rate of GLP on hydroxyl radical reached 63.78 ± 3.10%. It could be directly seen from Figure 6(c) that GLP showed strongly dose-dependent clearance against DPPH radicals. When the concentration was 1000 mg/L, the scavenging rates of GLP on DPPH radicals were 73.91 ± 3.53%.

### 3.7. Acute and Subchronic Toxicity Analysis

In the current study, the mice given continuous gavage for 10 days did not show any significant behavioral changes, including irritation, respiratory distress, abnormal movement, coma, or poisoning symptoms. At the same time, after 20 days of continuous gavage, there was no significant difference in body weights, inflammatory factor indexes, and serum biochemical indexes of mice in the NC groups of the three dose groups, indicating that GLP was actually a nontoxic substance (Table S2 and Table S3).

## 4. Discussion

A large number of reports have indicated that GLP is an active substance with functions of immunoregulation, antioxidation, antiaging, and antitumor [40–42]. However, the role of GLP in MODS model mice needs further research. In the past 20 years, great efforts have been made on the research of MODS. Researches have suggested that MODS is caused by a cascade-like enlarged systemic inflammatory response syndrome (SIRS) [5]. Based on the existing research, we chose animal models of MODS caused by injection of zymosan for experiments, owing to the model characteristics similar to the progression of the clinical MODS. Because of the properties of nonbiodegradable, the model is shown as the characteristics of the dual-phase delay. When zymosan is phagocytosed by macrophages, it causes a secondary blow, leading to continued inflammation in the body, thus inducing the MODS [43].

It has been reported that the oxygen free radicals are an important factor leading to the onset of MODS [44]. The lower activities and dysfunctions of antioxidant enzymes, such as SOD and CAT, can enlarge the generation of active oxygen, leading the oxygen free radicals to be the main pathogenic factor [45]. SOD and CAT can convert the most toxic superoxide ion free radicals into O_2_ and H_2_O_2_, thereby detoxifying superoxide ions and slowing the damage of free radicals [35, 36]. MDA is a product of lipid peroxidation, which can severely damage the cell membrane structure, and its content often incarnates the degree of lipid peroxidation in the body and indirectly reflects the degree of cell damage [46]. Our experimental results represented that the hepatic activities of SOD and CAT of mice in the MC groups were significantly reduced, and the MDA contents were significantly increased, which indicated that severe oxidative stress had been occurred in the body, causing tissue structure damage. Interestingly, after GLP intervention in different dose groups, the activities of SOD and CAT were significantly raised, which improved the body's ability to resist oxidation. Compared with other reports, Jin et al. has evaluated the structure and antioxidant activity of *Ulmus pumila* L polysaccharide (PPU) *in vitro*, and the results showed that selenization could improve the antioxidant capacity of polysaccharides, which also provided ideas for us to further improve the antioxidant capacity of GLP [47].

Zymosan-induced inflammation model has been widely used in the study of multiorgan failure. This model is also considered to be the most similar animal model to human MODS, because the reagent dose used is relatively uniform at home and abroad, and the MODS index changes caused by this model are similar to human clinical pathogenesis characteristics [48]. When zymosan were intraperitoneally injected into mice, they activated a variety of cells involved in the immune response, including macrophages, neutrophils, and natural killer cells. After activation, these cells can synthesize and secrete a series of cytokines associated with inflammatory response, such as TNF-*α*, IL-1*β*, IL-6, COX-2, and PGE2 [49]. TNF-*α* is mainly produced by monocytes and macrophages. As the earliest signal factor, TNF-*α* can initiate, amplify, and continue the systemic or local inflammatory response [50, 51]. When lipopolysaccharide enters the body, it will stimulate macrophages to produce a large amount of TNF and, then, induce a large number of inflammatory factors including IL-1*β* and IL-6 [51]. Besides, the significant rise of TNF-*α* levels in the liver can lead to the increased capillary permeability and further hypoxia in tissues and organs. At the same time, the levels of IL-6 and IL-1*β* also significantly increased, indicating that an inflammatory cascade effect was formed in the body, causing tissue damage, and the model was successfully constructed. Interestingly, the TNF-*α*, IL-1*β*, and IL-6 levels were significantly reduced after GLP intervention in different dose groups, suggesting that GLP can alleviate inflammation by reducing the levels of these important inflammatory factors. Additionally, PGE2 can increase the capillary permeability and aggravate edema caused by the penetration of other inflammatory cells. Therefore, inhibition of the production and release of PGE2 can effectively inhibit the development of inflammation [52]. The experimental results showed that the hepatic COX-2 and PGE2 levels treated with GLP at high dose were significantly lower than that in the MC groups, indicating that GLP could exert anti-inflammatory effects by inhibiting the expression levels of COX-2 and then inhibiting PGE2 synthesis.

Excessive inflammatory responses can cause hepatic cell necrosis and apoptosis which resulted in liver injury. When liver cells were damaged and lysed, the transaminase such as ALT and AST will be released in large quantities, resulting in a sharp increase in transaminase activities of serum [21]. AST activity is the most common indicator used for assessing liver injury [37]. At the same time, studies had found that excessive accumulation of TG and LDL-C can cause disorders in blood lipid metabolism and liver damage. We tested the biochemical indicators of mouse serum and found that the levels of ALT, AST, and ALP in the serum of mice in the MC groups were increased significantly, and the levels of TG and LDL-C were also enhanced, indicating that the mice had suffered severe liver damages [36]. However, after GLP intervention, the situation was relieved and liver damage was reduced. Therefore, we believe that GLP can protect the liver in MODS models. To further verify this conclusion, the histopathological observations of the liver were performed, and the results showed that the nucleus arrangement was more regular, cell structure was more normal, and liver injury was improved in the GLP dose group when compared with that in the MC groups. Combined with changes in inflammatory cytokines, GLP is believed to reduce the secretion rate of proinflammatory cytokines such as TNF-*α*, IL-1*β*, and IL-6, improve hepatic microcirculation failure, reduce hepatic apoptosis, and prevent liver injury and dysfunction. Our data are similar to those reported by Chen et al. [53] and Ishimoto et al. [54]. So we can conclude that GLP may be a potential therapeutic drug for the prevention and treatment of MODS, and its mechanism may be related to inhibiting oxidative stress response and reducing the level of key inflammatory factors *in vivo*.

The physiological activities of polysaccharides is closely related to its structures, and our structural identifications of GLP facilitate to understand the structure-activity relationship. Presently, the structural analysis of GLP showed that GLP mainly contained glucose with the bond types of -OH and C-O-C, being reported positive relationships with the antioxidant capacities, which was consistent with the results of our work [55, 56]. Furthermore, GLP has the basic framework of pyranose glucose, which is the premise to exert the antioxidant activity. Meanwhile, GLP has the substitution of glycolyl hexarbons, and only the branched side-chains can ensure the structural stability of the sugar chain screw, which can better guarantee the activities of GLP. The exploration of GLP structure lays a theoretical foundation for the next step of molecular mechanism research.

## 5. Conclusion

GLP could increase the level of antioxidant enzymes and reduce the production of lipid peroxides to effectively alleviate oxidative stress and enhance the body's antioxidant capacity. At the same time, GLP was able to improve the inflammatory response in MODS mice by inhibiting the levels of inflammatory factors and have certain protective effects on the liver. These findings might provide a theoretical basis for GLP to treat MODS.

## Data Availability

The data used to support the findings of this study are available from the corresponding author upon request.

## Abbreviations

ALT: Alamine aminotransferase
ALP: Alkaline phosphatase
Ara: Arabinose
AST: Glutamic oxalacetic transaminase
BHT: Butylated hydroxytoluene
CAT: Catalase
COX-2: Cyclooxygenase-2
FT-IR: Fourier-transform infrared
Fuc: Fucose
Gal: Galactose
GalA: Galacturonic acid
GalN: Galactosamine
Glc: Glucose
GlcA: Glucuronic acid
GlcN: Glucosamine
GLP: *Ganoderma lucidum* polysaccharides
GLPC: Gel permeation chromatography
HE: Hematoxylin-eosin
HDL-C: High-density lipoprotein cholesterol
HPLC: High-performance liquid chromatography
IL-1*β*: Interleukin-1*β*
IL-6: Interleukin-6
LDL-C: Low-density lipoprotein cholesterol
Man: Mannose
MODS: Multiple organ dysfunction syndrome
MDA: Malondialdehyde
MC group: Model control group
Mw: Weight-average molecular weight
Mn: Number-average molecular weight
NC group: Normal control group
NMR: Nuclear magnetic resonance
PGE2: Phenyl glycidyl ether 2
Rib: Ribose
Rha: Rhamnose
SOD: Superoxide dismutase
TNF-*α*: Tumor necrosis factor-*α*
TG: Triglyceride
Xyl: Xylose.

## Supplementary Materials

Supplementary 1 Table S1: molecular weight analysis of GLP.

Supplementary 2 Table S2: effects of different doses on the body weight of males mice for 20 days.

Supplementary 3 Table S3: effects of different doses on the serum index and liver inflammatory factor examination in male mice for 20 days.

## References

[1] Papathanassoglou E. D., Bozas E., Giannakopoulou M. D. (2008). Multiple organ dysfunction syndrome pathogenesis and care: a complex systems’ theory perspective. *Nursing in Critical Care*.

[2] Zheng Y., Zhu D. (2016). Molecular hydrogen therapy ameliorates organ damage induced by sepsis. *Oxidative Medicine and Cellular Longevity*.

[3] Barie P. S., Hydo L. J., Pieracci F. M., Shou J., Eachempati S. R. (2009). Multiple organ dysfunction syndrome in critical surgical illness. *Surgical Infections*.

[4] Martin G. S., Mannino D. M., Eaton S., Moss M. (2003). The epidemiology of sepsis in the United States from 1979 through 2000. *The New England Journal of Medicine*.

[5] Poggi C., Dani C. (2018). Sepsis and oxidative stress in the newborn: from pathogenesis to novel therapeutic targets. *Oxidative Medicine and Cellular Longevity*.

[6] Bota D. P., Melot C., Ferreira F. L., Ba V. N., Vincent J.-L. (2002). The multiple organ dysfunction score (MODS) versus the sequential organ failure assessment (SOFA) score in outcome prediction. *Intensive Care Medicine*.

[7] Steinberg S., Flynn W., Kelley K. (1989). Development of a bacteria-independent model of the multiple organ failure syndrome. *Archives of Surgery*.

[8] Goris R. J., Nuytinck J. K., Boekholtz W. K., Bebber I. P., Schillings P. H. (1986). Multiple-organ failure and sepsis without bacteria an experimental model. *Archives of Surgery*.

[9] Im K., Nguyen T., Do Shin K. L., Lee T. (2014). Appraisal of antioxidant and anti-inflammatory activities of various extracts from the fruiting bodies of *Pleurotus florida*. *Molecules*.

[10] Zhang J., Ma Z., Zheng L. (2014). Purification and antioxidant activities of intracellular zinc polysaccharides from *Pleurotus cornucopiae* SS-03. *Carbohydrate Polymers*.

[11] Lin L., Cui F., Zhang J. (2016). Antioxidative and renoprotective effects of residue polysaccharides from *Flammulina velutipes*. *Carbohydrate Polymers*.

[12] Liu G. Q., Zhang K. C. (2005). Mechanisms of the anticancer action of *Ganoderma lucidum* (Leyss. ex Fr.) Karst: a new understanding. *Journal of Integrative Plant Biology*.

[13] Chen H. S., Tsai Y. F., Lin S. (2004). Studies on the immuno-modulating and anti-tumor activities of *Ganoderma lucidum* (Reishi) polysaccharides. *Bioorganic & Medicinal Chemistry*.

[14] Bishop K. S., Kao C. H. J., Xu Y., Glucina M. P., Paterson R. R. M., Ferguson L. R. (2015). From 2000years of *Ganoderma lucidum* to recent developments in nutraceuticals. *Phytochemistry*.

[15] Tan X., Sun J., Xu Z. (2018). Effect of heat stress on production and *in-vitro* antioxidant activity of polysaccharides in *Ganoderma lucidum*. *Bioprocess and Biosystems Engineering*.

[16] Zhang H., Nie S., Cui S. W., Xu M., Ding H., Xie M. (2017). Characterization of a bioactive polysaccharide from *Ganoderma atrum*: re-elucidation of the fine structure. *Carbohydrate Polymers*.

[17] Ferreira I. C. F. R., Heleno S. A., Reis F. S. (2015). Chemical features of *Ganoderma* polysaccharides with antioxidant, antitumor and antimicrobial activities. *Phytochemistry*.

[18] Wang Y. Y., Khoo K. H., Chen S. T., Lin C. C., Wong C. H., Lin C. H. (2002). Studies on the immuno-modulating and antitumor activities of *Ganoderma lucidum* (Reishi) polysaccharides: functional and proteomic analyses of a fucose-containing glycoprotein fraction responsible for the activities. *Bioorganic & Medicinal Chemistry*.

[19] Xu S., Dou Y., Ye B. (2017). *Ganoderma lucidum* polysaccharides improve insulin sensitivity by regulating inflammatory cytokines and gut microbiota composition in mice. *Journal of Functional Foods*.

[20] Huang S. Q., Ning Z. X. (2010). Extraction of polysaccharide from *Ganoderma lucidum* and its immune enhancement activity. *International Journal of Biological Macromolecules*.

[21] Zhang J., Liu M., Yang Y. (2016). Purification, characterization and hepatoprotective activities of mycelia zinc polysaccharides by *Pleurotus djamor*. *Carbohydrate Polymers*.

[22] Zhu Z., Hu T., Wang Z. (2017). Anti-inflammatory and organ protective effect of insulin in scalded mods rats without controlling hyperglycemia. *The American Journal of Emergency Medicine*.

[23] Smirnoff N., Cumbes Q. J. (1989). Hydroxyl radical scavenging activity of compatible solutes. *Phytochemistry*.

[24] Li B., Zhang X. Y., Wang M. Z., Jiao L. L. (2015). Characterization and antioxidant activities of acidic polysaccharides from *Gynostemma pentaphyllum* (Thunb.) Markino. *Carbohydrate Polymers*.

[25] Zhang Y., Wang H., Wang P., Ma C. Y., He G. H., Rahman M. R. T. (2016). Optimization of PEG-based extraction of polysaccharides from *Dendrobium nobile* Lindl. and bioactivity study. *International Journal of Biological Macromolecules*.

[26] Gao Z., Liu X., Wang W. (2019). Characteristic anti-inflammatory and antioxidative effects of enzymatic- and acidic-hydrolysed mycelium polysaccharides by *Oudemansiella radicata* on LPS-induced lung injury. *Carbohydrate Polymers*.

[27] Sheng J. C., Yu F., Xin Z. H., Zhao L. Y., Zhu X. J., Hu Q. H. (2007). Preparation, identification and their antitumor activities *in vitro* of polysaccharides from *Chlorella pyrenoidosa*. *Food Chemistry*.

[28] Feng T., Wang K., Liu F. (2017). Structural characterization and bioavailability of ternary nanoparticles consisting of amylose, *α*-linoleic acid and *β*-lactoglobulin complexed with naringin. *International Journal of Biological Macromolecules*.

[29] Ma G., Yang W., Mariga A. M. (2014). Purification, characterization and antitumor activity of polysaccharides from *Pleurotus eryngii* residue. *Carbohydrate Polymers*.

[30] Tu W., Zhu J., Bi S. (2016). Isolation, characterization and bioactivities of a new polysaccharide from *Annona squamosa* and its sulfated derivative. *Carbohydrate Polymers*.

[31] Meng F., Li Q., Qi Y., He C., Wang C., Zhang Q. (2018). Characterization and immunoregulatory activity of two polysaccharides from the root of *Ilex asprella*. *Carbohydrate Polymers*.

[32] Meng M., Cheng D., Han L., Chen Y., Wang C. (2016). Isolation, purification, structural analysis and immunostimulatory activity of water-soluble polysaccharides from *Grifola frondosa* fruiting body. *Carbohydrate Polymers*.

[33] Liu H., Fan Y., Wang W. (2012). Polysaccharides from *Lycium barbarum* leaves: isolation, characterization and splenocyte proliferation activity. *International Journal of Biological Macromolecules*.

[34] Dore C., Alves M., Santos M., De Souza L., Baseia I., Leite E. (2014). Antioxidant and anti-inflammatory properties of an extract rich in polysaccharides of the mushroom *Polyporus dermoporus*. *Antioxidants*.

[35] Xu N., Ren Z., Zhang J. (2017). Antioxidant and anti-hyperlipidemic effects of mycelia zinc polysaccharides by *Pleurotus eryngii* var. *tuoliensis*. *International Journal of Biological Macromolecules*.

[36] Govindan S., Johnson E. E. R., Christopher J., Shanmugam J., Thirumalairaj V., Gopalan J. (2016). Antioxidant and anti-aging activities of polysaccharides from *Calocybe indica* var. APK2. *Experimental and Toxicologic Pathology*.

[37] Vuda M., D’Souza R., Upadhya S. (2012). Hepatoprotective and antioxidant activity of aqueous extract of *Hybanthus enneaspermus* against CCl_4_-induced liver injury in rats. *Experimental and Toxicologic Pathology*.

[38] Drotman R. B., Lawhorn G. T. (2008). Serum enzymes are indications of chemical induced liver damage. *Drug and Chemical Toxicology*.

[39] Wang X., Lan Y., Zhu Y. (2018). Hepatoprotective effects of *Auricularia cornea* var. *Li*. polysaccharides against the alcoholic liver diseases through different metabolic pathways. *Scientific Reports*.

[40] Kang Q., Chen S., Li S. (2019). Comparison on characterization and antioxidant activity of polysaccharides from *Ganoderma lucidum* by ultrasound and conventional extraction. *International Journal of Biological Macromolecules*.

[41] Xu Y., Zhang X., Yan X. H. (2019). Characterization, hypolipidemic and antioxidant activities of degraded polysaccharides from *Ganoderma lucidum*. *International Journal of Biological Macromolecules*.

[42] Liu Y., Li Y., Zhang W., Sun M., Zhang Z. (2019). Hypoglycemic effect of inulin combined with *Ganoderma lucidum* polysaccharides in T2DM rats. *Journal of Functional Foods*.

[43] Wang H. W., Yang W., Lu J. Y. (2013). N-acetylcysteine administration is associated with reduced activation of NF-kB and preserves lung dendritic cells function in a zymosan-induced generalized inflammation model. *Clinical Immunology*.

[44] Griffiths B., Anderson I. D. (2009). Sepsis SIRS and MODS. *Surgery*.

[45] Xu N., Gao Z., Zhang J. (2017). Hepatoprotection of enzymatic-extractable mycelia zinc polysaccharides by *Pleurotus eryngii* var *tuoliensis*. *Carbohydrate Polymers*.

[46] Jia R., Cao L., Xu P., Jeney G., Yin G. (2012). *In vitro* and *in vivo* hepatoprotective and antioxidant effects of *Astragalus* polysaccharides against carbon tetrachloride-induced hepatocyte damage in common carp (*Cyprinus carpio*). *Fish Physiology and Biochemistry*.

[47] Lee J. H., Lee Y. K., Chang Y. H. (2017). Effects of selenylation modification on structural and antioxidant properties of pectic polysaccharides extracted from *Ulmus pumila* L. *International Journal of Biological Macromolecules*.

[48] Li F., Lu J.-y., Liu Q., Wang H.-w., Guo H. (2013). Altered MARCH1 ubiquination-regulated dendritic cell immune functions during the early stage of zymosan-induced multiple organ dysfunction syndrome (MODS) in mice. *Immunology Letters*.

[49] Shibata H., Yoshioka Y., Ohkawa A. (2008). The therapeutic effect of TNFR1-selective antagonistic mutant TNF-*α* in murine hepatitis models. *Cytokine*.

[50] Tanno D., Akahori Y., Toyama M. (2014). Involvement of Gr-1 dull+ cells in the production of TNF-*α* and IL-17 and exacerbated systemic inflammatory response caused by lipopolysaccharide. *Inflammation*.

[51] Nardocci G., Martin A., Abarzúa S. (2015). Sepsis progression to multiple organ dysfunction in carotid chemo/baro-denervated rats treated with lipopolysaccharide. *Journal of Neuroimmunology*.

[52] Silva I. S., Nicolau L. A. D., Sousa F. B. M. (2017). Evaluation of anti-inflammatory potential of aqueous extract and polysaccharide fraction of *Thuja occidentalis* Linn in mice. *International Journal of Biological Macromolecules*.

[53] Chen Y. S., Chen Q. Z., Wang Z. J., Hua C. (2019). Anti-inflammatory and hepatoprotective effects of *Ganoderma lucidum* polysaccharides against carbon tetrachloride-induced liver injury in Kunming mice. *Pharmacology*.

[54] Ishimoto Y., Ishibashi K. I., Yamanaka D. (2018). Protection against gut inflammation and sepsis in mice by the autodigested product of the Lingzhi medicinal mushroom, *Ganoderma lingzhi* (*Agaricomycetes*). *International Journal of Medicinal Mushrooms*.

[55] Wang M., Zhu P., Zhao S. (2017). Characterization, antioxidant activity and immunomodulatory activity of polysaccharides from the swollen culms of *Zizania latifolia*. *International Journal of Biological Macromolecules*.

[56] Ren Z., Li J., Xu N. (2017). Anti-hyperlipidemic and antioxidant effects of alkali-extractable mycelia polysaccharides by *Pleurotus eryngii* var. *tuolensis*. *Carbohydrate Polymers*.

