# Supplementary material for: Characterization and Hepatoprotections of Ganoderma lucidum Polysaccharides against Multiple Organ Dysfunction Syndrome in Mice
Source: Oxid Med Cell Longev. 2021 Feb 3;2021:9703682. doi: 10.1155/2021/9703682 (PMC7876828; doi:10.1155/2021/9703682)
Supplement: Supplementary 1 — Table S1: molecular weight analysis of GLP. [file 9703682.f1.docx]

Table S1 Molecular weight analysis of GLP

| Sample | Molecular weights |
| --- | --- |
| Mw (Da) | 8309 |
| Mn (Da) | 4211 |
| Mw/Mn | 1.97 |

GLP: *Ganoderma lucidum* polysaccharides; Mw: weight-average molecular weight; Mn: number-average molecular weight.
