# Supplementary material for: Characterization and Hepatoprotections of Ganoderma lucidum Polysaccharides against Multiple Organ Dysfunction Syndrome in Mice
Source: Oxid Med Cell Longev. 2021 Feb 3;2021:9703682. doi: 10.1155/2021/9703682 (PMC7876828; doi:10.1155/2021/9703682)
Supplement: Supplementary 2 — Table S2: effects of different doses on the body weight of males mice for 20 days. [file 9703682.f2.docx]

Table S2 Effects of different doses on the body weight of males mice for 20 days.

| Groups |  | Body weight (g) |  |
| --- | --- | --- | --- |
|  | Initial |  | Final |
| NC | 22.47 ± 1.96 |  | 33.23 ± 2.01 |
| 900 mg/kg | 23.34 ± 1.65 |  | 30.66 ± 1.87 |
| 1200 mg/kg | 25.12 ± 2.01 |  | 35.36 ± 1.79 |
| 1500 mg/kg | 25.05 ± 1.85 |  | 34.64 ± 1.96 |

The values were reported as the Mean ± S.D. of ten mice in each group. NC: normal control groups
