# Supplementary material for: Characterization and Hepatoprotections of Ganoderma lucidum Polysaccharides against Multiple Organ Dysfunction Syndrome in Mice
Source: Oxid Med Cell Longev. 2021 Feb 3;2021:9703682. doi: 10.1155/2021/9703682 (PMC7876828; doi:10.1155/2021/9703682)
Supplement: Supplementary 3 — Table S3: effects of different doses on the serum index and liver inflammatory factor examination in male mice for 20 days. [file 9703682.f3.docx]

Table S3 Effects of different doses on the serum index and liver inflammatory factor examination in males mice for 20 days.

| Groups | Serum index | | | | |  | Inflammatory factor analysis | | |
| --- | --- | --- | --- | --- | --- | --- | --- | --- | --- |
|  | ALT  (U/L) | AST  (U/L) | ALP  (U/L) | TG  (mmol/L) | TP  (g/L) |  | TNF-α  (pg/mL) | IL-6  (pg/mL) | IL-1β  (pg/mL) |
| NC | 32.42 ± 2.45 | 54.18 ± 2.09 | 28.41 ± 1.71 | 1.90 ± 0.07 | 69.32 ± 1.88 |  | 28.69 ± 1.67 | 28.71 ± 1.46 | 17.92 ± 1.31 |
| 900 mg/kg | 28.49 ± 1.97 | 51.0 2± 1.68 | 26.33 ± 1.49 | 1.52 ± 0.09 | 65.79 ± 2.67 |  | 22.17 ± 1.35 | 27.69 ± 2.05 | 18.36 ± 1.91 |
| 1200 mg/kg | 27.55 ± 2.01 | 51.90 ± 2.31 | 27.57 ± 1.60 | 1.88 ± 0.03 | 66.82 ± 2.33 |  | 25.83 ± 1.95 | 28.26 ± 1.72 | 14.13 ± 1.22 |
| 1500 mg/kg | 30.29 ± 1.57 | 50.52 ± 1.55 | 28.22 ± 2.07 | 2.01 ± 0.085 | 68.11 ± 2.03 |  | 29.48 ± 1.88 | 31.45 ± 1.82 | 21.64 ± 1.43 |

The values were reported as the Mean ± S.D. of ten mice in each group. NC: normal control groups; ALT: alamine aminotransferase; ALP: alkaline phosphatase; TG: triglyceride; TP: total protein; TNF-α: tumor necrosis factor-α; IL-6: interleukin-6; IL-1β: interleukin-1β.
